# Disparities in Healthcare Access, Education, and Geographical Factors That Affect Surgical Outcomes in Penile Cancer

**DOI:** 10.7759/cureus.30068

**Published:** 2022-10-08

**Authors:** Juan Carlos Angulo-Lozano, Luisa Fernanda Sánchez Musi, Jose Garcia Garcia

**Affiliations:** 1 School of Medicine, Universidad Anahuac Mexico, Huixquilucan, MEX; 2 Urology Department, Hospital General de Mexico, Mexico City, MEX; 3 Health and Social Behavior, Harvard School of Public Health, Boston, USA

**Keywords:** lack of access to primary care, partial penectomy, total penectomy, disparities, urologic cancer

## Abstract

Objectives: To establish the level of access to healthcare, education, social and geographical factors predisposing a negative surgical outcome and higher mortality rate in patients with penile cancer.

Methods: This is a retrospective, longitudinal and analytical study. Ninety-three medical records of adult male patients diagnosed with penile cancer were reviewed. Fisher’s exact test was performed to determine the association between the level of healthcare, social and geographical factors, and the outcomes for penile cancer.

Results: Patients without primary care access had a higher chance of having lymphovascular invasion at the time of diagnosis (OR=37.5, P<0.0001), a higher mortality rate at 24 months after diagnosis (OR=19.2, P=0.005), a lack of high school diploma or equivalent (OR=3.8, P=0.049) and a higher likelihood of referral from a provincial hospital (OR=10.1, P<0.0001). Patients without a favorable surgical outcome (radical penectomy) were more likely to have been referred from a provincial hospital (OR=6.8, P<0.0001) and not have access to a primary care center (OR=149.5, P<0.0001), a tertiary care center (OR=20.7, P=0.003), and a high school diploma (OR=7.9, P=0.004).

Conclusions: The lack of access to primary care is strongly associated with vascular invasion at diagnosis, lower educational level, a referral from provincial zones, and a higher mortality rate at 24 months. Patients who did not have access to primary and tertiary care, a high school diploma, and were referred from the province were more likely to have a radical penectomy.

## Introduction

Penile carcinoma is a rare condition that mainly presents in older patients, with a mean age range at diagnosis within the sixth decade of life (68 years) [1-4]. Of these cases, around 95% are of squamous cell (SCC) origin, making it the most common type, followed by some reports of sarcoma, melanoma, and basal cell carcinoma [5,6]. Establishing a therapeutic approach requires special infrastructure and prior microscopic analysis of a biopsy specimen to confirm the diagnosis and determine the degree of invasion [7]. This process should necessarily be carried out in specialized care units. 

Health care mentioned in the variables of this study is divided into three levels: primary care consisting of general and family practitioners, secondary consisting of basic specialized care, and tertiary consisting of specialized and subspecialized care. Treatment and follow-up should be administered at specialized care units, and it is dependent on the type of neoplasm and degree of invasion. It ranges from minimally invasive approaches to surgical techniques such as circumcision, glansectomy, and partial or total penectomy [3,8-10]. For this reason, it is crucial to identify potential risk factors and diagnose patients at an early stage of the disease to refer them to specialized care. 

Among the identified risk factors for developing penile cancer, the human papilloma virus (HPV) is the most notably recognized. As such, precursor lesions and penile SCC variants have been recently divided into two major categories: related and non-related to HPV [11]. Nevertheless, other factors can also elevate the risk of developing this condition, such as a history of phimosis, number of sexual partners, presence of circumcision, and tobacco exposure [11,12]. Circumcision, male vaccination of HPV, early treatment of phimosis, smoking cessation, and hygiene practices are some of the potential techniques for preventing penile cancer. 

Interestingly, Mexico has an incidence rate of 0.91 per 100,000 males with a mortality rate of 0.2 per 100,000, with the mean average age at diagnosis of 68 years, representing 0.17% of cancer deaths [13]. However, although the incidence is relatively low, patient outcomes tend to be radical, and the prognosis is usually poor. For this reason, patients with potential risk factors recognized at their primary care clinic and those identified with this diagnosis should be promptly referred to specialized tertiary care units. In this way, low levels of schooling and lack of access to primary (and consequently tertiary) care have a negative impact on the outcome of patients diagnosed with penile carcinoma. Preventive measures would necessitate a thorough cost-benefit analysis and significant changes in health policy. 

Since this is a retrospective study, limitations should be acknowledged such as the absence of data on probable confounding factors that may be missing and was not reported or gathered, differential losses on follow-up also could bias the results and interpretation of this study. Furthermore, a potential systematic error can be the difficulty to identify an appropriate exposed cohort and appropriate comparison group.

This article was previously posted to the Research Square preprint server on May 9th, 2022.

## Materials and methods

This article has been reviewed and approved by the ethics research committee of Mexico General Hospital, as it complies with the general ethical code of the Mexico General Hospital, with the approval number HGM-040122-2300.

This is a retrospective, longitudinal and analytical study. Ninety-three medical records of adult male patients diagnosed with penile cancer were reviewed, diagnosed by biopsy analyzed by the Pathology Department of the Hospital General de Mexico "Dr. Eduardo Liceaga," a specialized hospital in Mexico City. The inclusion criteria considered for this study were: male adults of 18 years or older at the date of diagnosis, diagnosis of penile cancer by biopsy, and patients who were treated at the Hospital General de Mexico. Exclusion criteria was: pathologic reports inconsistent with penis cancer. One hundred sixty-eight medical files were examined, and 75 patients were not included in the analysis because of biopsies with dysplastic or inconsistent diagnoses with penile cancer.

Fisher’s exact test was performed to determine the association between access to primary and tertiary care centers and the following factors: pathological diagnosis of vascular invasion, death of cancer-related causes at 24 months, a referral from provincial zones, and whether the patient had a high school diploma. Additionally, we analyzed the association between total resection of the penis with access to primary and tertiary care, referral status from provincial zones, and whether the patient had a high school diploma. We reported odds ratios (OR) for statistically significant associations and used a Haldane-Anscombe correction to remove bias from the estimator. 

## Results

A total of 52 patients (55.9%) had access to a primary care center, nine (9.67%) had access to a tertiary care center, zero patients (0%) had health insurance, 15 (55.9%) had a High School diploma or equivalent, and 39 (41.9%) were referred from a province hospital. Regarding the risk factors, 52 (55.9%) had a history of smoking, 93 (100%) were uncircumcised, 77 (82.1%) were HPV negative on histopathological report and 16 (17.2%) were HPV positive. 

SCC was predominant, representing 86% of the cases for penile carcinoma, and 14% were reported different variants. Seventeen (18.3%) had lymphovascular invasion (Table 1). At 24 months after diagnosis, 79 (84.9%) patients were alive. Treatment approaches in the study group are outlined in Table 2.

**Table 1:** Epidemiological characteristics of the study group and causes of death SD: Standard deviation HPV: Human papilloma virus

Epidemiological Characteristics of the study group (n = 93)	
Age (years ± SD)	57.87 ± 12.93
Smoking status (%)	52 (55.9%)
Uncircumcised (%)	93 (100%)
HPV + (%)	16 (17.2%)
Squamous Cell Carcinoma (%)	80 (86%)
Lymphovascular invasion (%)	76 (81.7%)
Access to primary care center	52 (55.9%)
Access to tertiary care center	9 (9.67%)
Health Insurance	0 (0%)
High School diploma or equivalent	15 (16.1%)
Referred from a province hospital	39 (41.9%)
Cause of death	Total = 14
Cancer-related causes	6 (42.8%)
Other causes	6 (42.8%)
Chemotherapy toxicity	2 (14.4%)

**Table 2:** Treatment modality

Surgical treatment	Total = 93	Radical inguinal Lymphadenectomy (n=22)
Radical penectomy (%)	45 (48.4%)	15 (68.1%)
Partial penectomy (%)	34 (36.6%)	7 (31.9%)
Pharmacologic treatment (%)	14 (15.2%)	0 (0%)
Adjuvant treatment	Total = 93	Death at 24 months (n=14)
Chemotherapy + Radiotherapy	12 (12.9%)	8 (57.1%)
No adjuvant therapy	81 (87.1%)	6 (42.9%)

Surgical approaches included radical and partial penectomy, out of which 45 patients (48.4%) had radical penectomy, 34 (36.6%) had a partial penectomy, and 14 (15.1%) of the patients did not have surgical management. Additionally, 21 patients (22.5%) had radical inguinal lymphadenectomy. On the other hand, adjuvant approaches included chemotherapy and adjuvant radiotherapy. Concerning these approaches, 81 patients (87.1%) did not receive adjuvant chemotherapy, and 12 (12.9%) received adjuvant chemotherapy and radiotherapy. 

Fisher’s exact test with Haldane-Anscombe correction showed a statistically significant association in patients without primary care access and a higher likeliness of having lymphovascular invasion at the time of diagnosis (OR=37.5, P<.0001), a higher mortality rate at 24 months after diagnosis (OR=19.2, P=.005), a lack of high school diploma or equivalent (OR=3.8, P=.049) and a higher likelihood of referral from a provincial hospital (OR=10.1, P<.0001). Similarly, patients without tertiary care access were more likely to have lymphovascular invasion at the time of penile cancer diagnosis (OR=196.3, P<.0001) and to be referred from a provincial hospital (OR=15.6, P=.009).

Patients without tertiary care access did not show a statistically significant difference in mortality at 24 months after diagnosis (P=0.76) and whether they had a high school diploma (P=0.66) (Table 3). The relationship between total resection of the penis and probable predisposing factors is presented in Table 4.

**Table 3:** Relationship between access to primary or tertiary care and related factors

Lack of access to primary care	OR (95% CI)	P value
Lymphovascular invasion	37.5 (2.1 – 647.6)	<0.0001
Higher mortality rate at 24 months	19.2 (1.04 – 352.1)	0.005
No high school diploma	3.8 (0.9 – 14.5)	0.049
Likelihood of referral from a provincial hospital	10.1 (3.8 – 26-6)	<0.0001
Lack of access to tertiary care	OR (95% CI)	P value
Lymphovascular invasion	196.3 (10.3 – 3713.4)	0.0004
Higher mortality rate at 24 months	1.5 (.08 – 30.1)	0.76
No high school diploma	0.62 (0.07 – 5.4)	0.66
Likelihood of referral from a provincial hospital	15.6 (1.1 – 292.5)	0.009

**Table 4:** Relationship between radical penectomy and predisposing factors

Radical Penectomy	OR (95% CI)	P value
Lack of access to primary care	149.5 (28.5 – 783.3)	<0.0001
Lack of access to tertiary care	20.7 (1.2 – 388.1)	0.003
No high school diploma	7.9 (1.6 – 37.7)	0.008
Referred from a provincial hospital	6.8 (2.7 – 17.3)	<0.0001

Interestingly, patients without a favorable surgical outcome (radical penectomy) were more likely to have been referred from a provincial hospital (OR=6.8, P<.0001) and to not have access to a primary care center (OR=149.5, P<.0001), a tertiary care center (OR=20.7, P=0.003), and a high school diploma (OR=7.9, P=0.004). 

## Discussion

Low levels of schooling and lack of access to primary (and consequently tertiary) care can have a negative impact on the outcome of patients diagnosed with penile carcinoma. Because of the disease's rarity, data collection and standardization in clinical practice have been limited, especially in Latin American countries. As such, this study aimed to determine the social and geographical factors that could predispose to a late diagnosis of penile cancer, an advanced stage, and an unfavorable surgical outcome. Interestingly, almost half of our cohort had a radical penectomy as the primary and curative treatment. This raises questions about which factors predispose patients to a delayed diagnosis leading to unfavorable outcomes. 

Several findings in this study are worth discussing. Firstly, as it has been mentioned before, almost half of the patients had a radical penectomy. This study demonstrated a relationship between total resection of the penis and the lack of access to primary care, tertiary care, lower level of education, and patients who were referred from provincial zones. This radical outcome may negatively impact the patient's psychological and sexual aspects, resulting in a lower quality of life. On the other hand, the mean age (57 years) at diagnosis was significantly lower than what has previously been reported in the literature (68 years) [4]. Remarkably, all the patients in the study were uncircumcised; in Latin America, the circumcision prevalence ranges between 10-31%. This factor could explain the higher incidence of penile cancer in Latin America and countries with a low circumcision prevalence, as uncircumcised men present a higher incidence of invasive penile cancer [14,15]. 

It has previously been reported that developing countries show disparities in outcomes due to several factors, including lack of primary care services, low educational level, misdiagnosis, advanced tumor stage at diagnosis, follow-up due to labor circumstances, and delayed referral due to lack of specialized services in marginalised communities [16,17]. Noticeably, the results of this study support these statistics because almost half of our patients were referred from province communities delaying their diagnosis and treatment. Conversely, while HPV has been strongly associated with penile cancer incidence, our study reports a relatively low prevalence of HPV positivity by pathology [12]. This finding may be explained by the fact that we did not perform serological studies and relied solely on histopathological findings suggestive of HPV infection. 

The findings in this study suggest that those who did not have access to health centers were more likely to present risk factors known to have undesirable outcomes like higher mortality rate, total resection of the penis, and vascular invasion at the time of diagnosis. It is necessary to eradicate disparities among social groups so that every person can access primary health centers to improve the mortality rate and quality of life of patients with penile cancer. 

Sociodemographic factors such as race (Hispanic and African American men) have been previously associated with a younger age presentation, higher stage of disease at diagnosis, and worse survival rates [1]. However, to our knowledge, no studies have associated factors such as access to primary care, tertiary care, and referral from provincial units to surgical outcomes and survival outcomes. It is particularly important to mention that the data used in this study was gathered at a public hospital in Mexico City and thus, can only be extrapolated to countries that share similar demographic characteristics such as race, socioeconomic and geopolitical factors (e.g., some Latin American countries, among other developing countries). Moreover, it is worth mentioning that the data must be interpreted with caution because of the limited number of patients in our study group and the fact that access to secondary centers was not considered. 

It is essential to highlight the relevance of circumcision as a preventive method for penile cancer, besides the lower risk of infection of HPV since developing countries have a low circumcision prevalence. HPV vaccines in men could help reduce the incidence of penile malignancies, but there are still no prospective studies to conclude this recommendation although is biologically plausible. We also recommend immediate referral to tertiary care centers when suspecting a penile cancer lesion or premalignant lesions to prevent further delay in treatment and correct pathological reporting and therapy. Periodic evaluation and proper dose of chemotherapy, with adverse effects surveillance and serum concentration of the drug should also be considered since a significant proportion of the population died because of chemotherapy toxicity.

## Conclusions

Developing countries show disparities in outcome due to several factors, including lack of primary care services, misdiagnosis, advanced tumor stage at diagnosis, follow-up due to labor circumstances, and delayed referral due to lack of specialized services in marginalised communities. The lack of access to primary care is strongly associated with vascular invasion at diagnosis, lower educational level, a referral from provincial zones, and a higher mortality rate at 24 months. Patients who did not have access to primary and tertiary care, a high school diploma, and were referred from the province were more likely to have a total resection of the penis. 
